# Costly Advertising and the Evolution of Cooperation

**DOI:** 10.1371/journal.pone.0067056

**Published:** 2013-07-08

**Authors:** Markus Brede

**Affiliations:** Department of Electronics and Computer Science, University of Southampton, Southampton, Hampshire, United Kingdom; University of Maribor, Slovenia

## Abstract

In this paper, I investigate the co-evolution of fast and slow strategy spread and game strategies in populations of spatially distributed agents engaged in a one off evolutionary dilemma game. Agents are characterized by a pair of traits, a game strategy (cooperate or defect) and a binary ‘advertising’ strategy (advertise or don’t advertise). Advertising, which comes at a cost 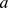, allows investment into faster propagation of the agents’ traits to adjacent individuals. Importantly, game strategy and advertising strategy are subject to the same evolutionary mechanism. Via analytical reasoning and numerical simulations I demonstrate that a range of advertising costs exists, such that the prevalence of cooperation is significantly enhanced through co-evolution. Linking costly replication to the success of cooperators exposes a novel co-evolutionary mechanism that might contribute towards a better understanding of the origins of cooperation-supporting heterogeneity in agent populations.

## Introduction

Cooperative behaviour - acting for the benefit of the group even if not in the immediate interest of the individual - is common in life. Examples are found in many simple and more complex organisms, ranging from bacteria in microfilms [1] up to humans and human society (see e.g. [2]). Explaining the emergence and sustainability of cooperation has attracted considerable interest over the last decades. Previous approaches typically use the framework of evolutionary game theory [3] that describes the spread of strategies in populations of individuals engaged in prototypical dilemma situations. Reproductive success is determined by payoffs which depend on an individual’s strategy and on the strategies of its interaction partners.

One of the most often studied dilemmas in this context is the prisoner’s dilemma. Two individuals are simultaneously faced with a choice between two options, “C” (for cooperate) and “D” (for defect). Mutual cooperation is rewarded with a payoff of 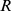. Defectors playing against cooperators receive the temptation to defect 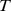 while cooperators are paid the “sucker’s” payoff 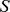 in these interactions. Last, mutual defection results in a payoff 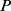, the punishment for mutual defection, for both players. Payoffs are ranked 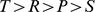 and 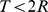 such that irrespective of an opponent’s choice an individual is best off by playing 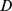. Hence the Nash equilibrium is 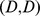 with a group payoff of 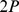 which is inferior to the social optimum of 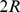 that could be achieved by playing 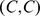. How then can cooperative behaviour be explained?

An approach that has found much attention in recent years is to consider evolutionary games in structured populations [4]. In this way, for instance, a spatially distributed population can support cooperation [5]. The basic cooperation-supporting mechanism is network reciprocity (see [6] for a classification of cooperation-supporting mechanisms), i.e. strategies assort in space such that clusters of cooperators can shield cooperation against the invasion of defection. The basic findings of [5] for the prisoner’s dilemma have been extended in many ways, e.g. by considering the effects of asynchronicity [7], various forms of noise [8]–[11], and payoff structures other than the prisoner’s dilemma game [12], [13].

More recently, also the evolution of cooperation on complex networks [14]–[16] has become a major field of study. The prevailing finding is that heterogeneity in network structure can strongly enhance the support for cooperation. This effect is due to the role of hub nodes as cooperation leaders [15], [17]. Hub nodes have many more opportunities to play the game (and hence generate payoff) than average nodes. Consequently, they tend to impose their strategies on adjacent nodes. Then, if a hub node was a defector, it would quickly undermine its position by surrounding itself with defectors, whereas it would reinforce its position when following a cooperate strategy. This basic effect of heterogeneity on cooperation can also be observed on regular or spatial networks when other heterogeneity in agent properties is introduced. Examples of this type of model are models of learning and teaching [18]–[20] or models that consider differential abilities of agents to evaluate [21] or generate payoff [22]–[24].

Whether assuming a scale-free network topology as in [15], a distribution of learning and teaching abilities as in [18], or quenched stochasticity in payoff structures as in [22], all the above studies presuppose a fixed heterogeneous system structure. Such an assumption can be reasonable if (e.g. environmental) processes unrelated to the evolutionary dilemma game shape system structure. However, it remains an important question to investigate how system structure and strategies can co-evolve to create the dynamic patterns that allow for cooperation to survive (see [25] for a review on that topic). Most prominently this question has been addressed in the context of adaptive networks and cooperation [17], [26]–[28]. Other studies have investigated mechanisms for the coevolution of teaching or learning abilities and cooperation [29]–[31]. This work typically relies either on reasonable ad-hoc rules [30], [31] or on a dynamics of system structure that is similar to Hebbian learning [32]: i.e. what is successful remains (as in the case of co-evolutionary network models [17], [26]–[28]) or becomes stronger (as e.g. in the context of [29]). Whereas such a combined dynamics can explain the co-evolution of strategies and cooperation-supporting structure, the dynamics of system structure is not subject to an evolutionary dynamics itself. Previous models like [17], [26]–[31] might thus be suitable in the context of human learning (or for any types of more sophisticated agents), but would be problematic in the context of very simple biological organisms.

To explain this point in more detail, consider a model of teaching and learning developed in [18]. The study investigates a setup in which two types of agents (e.g. teachers and learners) are subject to an evolutionary prisoner’s dilemma on a 2d spatial grid. Agent types are assumed to be fixed and cooperation is supported by the implied heterogeneity in strategy adaptation speed. Now consider a scenario in which agents’ teaching/learning abilities (or ‘advertising’ abilities as I will call them subsequently) are passed on together with their game strategies. What would be observed is that the teaching trait will spread in the population, thus reducing heterogeneity and hence removing the support for cooperation. This raises the question: Can evolutionary processes that govern the dynamics of both strategy and teaching trait give rise to the necessary heterogeneity to support cooperation? In this paper I will follow previous studies as [33], [34] that modelled individual trait selection in the prisoner’s dilemma game to address this topic. I demonstrate that the answer to this question is yes, but only provided that the advertising trait is costly and costs of advertising are within a certain range.

Subsequently I consider ‘advertising’ as an agent-specific ability to enhance its chances of strategy propagation. This may be understood in a social context as an individuals persuasiveness (or effort to persuade others to imitate it). In a biological context ‘advertising’ may be linked to an investment into replication that comes at a cost, or may be interpreted as a form of signalling that enhances an individual’s chance of being imitated. However, note a crucial difference to models of cheaptalk, green beard-type signalling and the evolution of cooperation [35]–[37]. The present model does not allow sophisticated agents that have the ability to play strategies that discriminate between game partners’ signals. Rather, signals promote the replication of strategies when present.

The organization of the paper is as follows. Section 0 starts with a description of the model and the typical setup of simulation experiments. Section 0 presents analytical results for the well-mixed case and then proceeds with a numerical analysis of the evolutionary game in space. The generality of results and the wider context are analyzed at the end of the results section and in the final section of the paper.

## Methods

I consider a set of 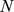 agents distributed on a 2d spatial 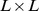 square lattice with periodic boundary conditions. Adjacency relationships are defined by von Neumann neighbourhoods. Agents are engaged in the one-off prisoner’s dilemma, play pure strategies, either 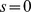 (for defect) or 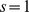 (for cooperate), and receive payoffs depending on game outcomes. Following a large part of the literature I parametrize the prisoner’s dilemma via
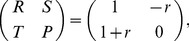
(1)such that the parameter 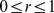 classifies the dilemma strength.

Additional to game strategies, agents have a trait 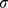 that determines their ‘advertising’ strategy. Hence, four possible strategy combinations: advertising cooperators (CA), non-advertising cooperators (C), advertising defectors (DA) and non-advertising defectors (D) are possible. An agent with 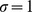 advertises, such that it has a by an amount 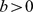 enhanced chance of being selected as a reference agent for strategy updating. Agents with 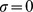 still have a chance of being selected as a reference, but this chance is smaller than the chance of being selected as an advertiser. Advertising is costly and an agent that advertises will have a cost 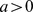 deducted from its payoff before strategy updating.

More specifically, the following algorithm is implemented for the evolution of game strategy 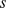 and advertising trait 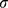:

- In a typical experiment the system is seeded with a random allocation of 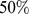 cooperators and 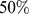 defectors. Both cooperators and defectors are equally likely to advertise or not. In some cases, in particular for large benefit of advertising, otherwise stable phases can not evolve out of randomly allocated initial conditions. In such cases I initialize simulations by a correlated arrangement of the four strategies such that like types cluster. Long term solutions do not depend on specifics of these initial arrangements.
- A focus agent 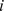 is chosen at random. Amongst the focus agent’s neighbours a reference agent is chosen probabilistically such that agents with the advertising strategy trait have an enhanced chance of being selected by the focus agent 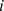, i.e.
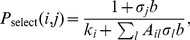
(2)where 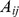 is the adjacency matrix of the contact network and 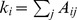 is the degree of node 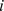.
- Game payoffs 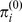 and 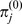 of both the focus and the reference agent are calculated from games they play against all of their respective neighbours. Following this advertising costs are deducted if applicable, i.e.

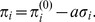
(3)In a next step strategies are updated according to Fermi-pairwise updating. Accordingly, with probability
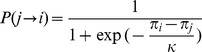
(4)agent 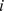 copies the strategy traits (both game and advertising strategies) from agent 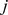. The parameter 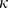 in Eq. (4) gives the intensity of noise in strategy updating and is set to 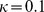 for all of the following simulation experiments.
- Steps 2,3, and 4 are iterated till a quasistationary state is reached and then equilibrium concentrations of the four strategy combinations: advertising cooperators 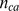, non-advertising cooperators 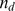, advertising defectors 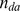, and non-advertising defectors 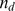 are determined as averages over a sufficient number of further iterations.

In all following experiments system sizes from 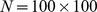 up to 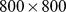 have been considered and system sizes were increased as required close to transition points.

The above model setup differs slightly from the setup of the orginal model of learning and teaching developed in [18] in which learning or teaching abilities are included as an agent-specific prefactor in the strategy transmission rates in Eq. (4). The purpose of the present setup is to make the role of advertising more explicit in the way agents select reference partners. Very similar results to the ones presented below can be obtained for models in which advertising is directly included as a prefactor to Eq. (4) and the probabilistic reference partner selection step is omitted.

## Results

### The Advertising Game

To understand the effect of advertising in combination with an evolutionary dilemma game, it is instructive to gain insights into the incentives for advertising in a well-mixed population. Let us consider a population composed of two types of agents, ‘advertisers’ (concentration 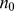) and non-advertisers (concentration 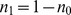). Payoff differences are then determined by the cost of advertising 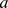 and I assume that strategy concentrations in the population evolve according to Eq.s (2) and (4). In the limit of large systems, the evolution of concentrations is governed by
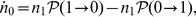
(5)where
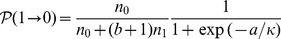
(6)and
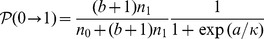
(7)are the transition probabilities that an advertiser changes its strategy to non-advertising or vice versa. The first factors in Eq.s (6) and (7) give the probability of selecting an agent of the other type as a reference and the second factors give the probabilities of adopting the other types’ strategy once the type has been selected as a reference. Combining Eq.s (5), (6) and (7) and straightforward manipulation gives a criterion for a critical cost of advertising 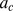


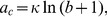
(8)such that advertising is not viable in a well-mixed population if 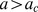 and dominates the entire population if 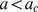.

A viscous population structure does not give any additional benefit for either advertisers or non-advertisers and only slows down the diffusion of strategies. Accordingly, one would expect the criterion (8) to hold on spatial lattices as well. This has been confirmed by simulation experiments (data not shown).

### Advertising and Cooperation in Well-mixed Populations

Consider the coevolution of advertising trait 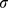 and game strategy 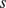 in a well-mixed population. In this setup two regimes must be distinguished. For cheap advertsing (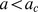) it can easily be shown that irrespective of the composition of the population net transition rates from any species in the population to advertising defectors are positive. Hence, 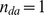 is a stable equlibrium point and cooperation is not sustainable. For expensive advertising 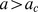 net transition rates from any species to non-advertising defectors are positive and hence 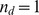 is a stable fixed point. Similar arguments show that for 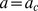 any composition of advertising and non-advertising defectors with 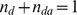 is a stable equilibrium point. Unsurprisingly, one concludes that advertising cannot facilitate stable cooperation in well-mixed populations.

### Results in Structured Populations

The above arguments change in structured populations, on which network reciprocity favours positive assortment of strategies. When network reciprocity is present, it is advantageous for cooperators to surround themselves with the same strategy. Hence there is a benefit to a cooperator to invest in ‘advertising’ that surpasses the threshold cost 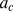 obtained from the cost-benefit analysis of the advertising game in the absence of a dilemma situation. Clearly also, this benefit of cooperators of surrounding themselves by like types is limited. One thus also expects an upper threshold cost of advertising 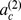 such that advertising for cooperators is no longer viable for 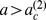.

The situation is different for defectors. Defectors don’t gain from surrounding themselves with like types. Hence, the threshold for the viability of advertising for defectors is the same as for the advertising game alone, i.e. given by Eq. (8). Both arguments let one surmise that there must be a range of advertising costs 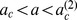 such that advertising is profitable for cooperators but not to defectors.

Spatial arrangements of strategies add a further dimension to the problem. Consider the range of costs 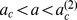 in which advertising is viable for cooperators, but not for defectors. Defectors close to cooperators can achieve very high payoffs, but payoffs of defectors surrounded by other defectors are poor. Similar to results on volunteering [38], [39], this leads to a cyclical dominance between strategies that allows for coexistence in spatial settings. In particular, in ‘tough’ games with 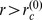, advertising defectors can outcompete advertising cooperators. However, the cost of advertising can disadvantage advertising defectors in direct competition with non-advertising defectors. Further, advertising cooperators may be able to invade groups of non-advertising defectors, when the cost of advertising is not too large, thus creating a cyclical dominance. For large 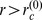 non-advertising cooperators never play a role: They are always outcompeted by all other strategies and are not expected to be found in the population.

**Figure 1:**
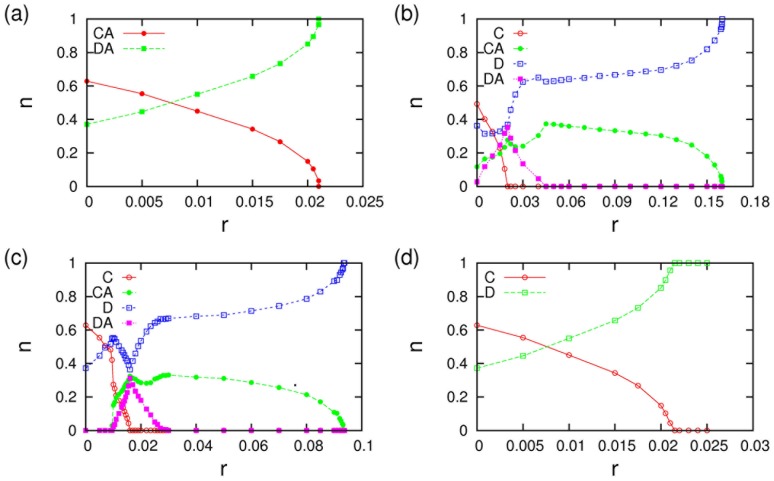
Dependence of stationary strategy concentrations of 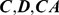 and 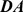 on 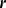 for an advertising benefit of 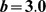 and 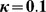. (a) Cost of advertising 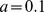: 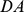 and 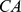 dominate. (b) 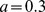. All four strategies coexist until C dies out at 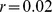. CA, D and DA coexist until DA dies out at 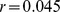. Finally, CA coexists with D and CA dies out at 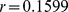 (c) 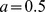. For 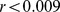 no advertising is found. All four strategies coexist in varying proportions for 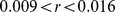. For 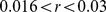 CA and DA and D coexist. For 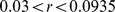 CA and D coexist. (d) 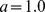. No advertising can be sustained. See also Fig. 2 for a full phase diagram.

**Figure 2:**
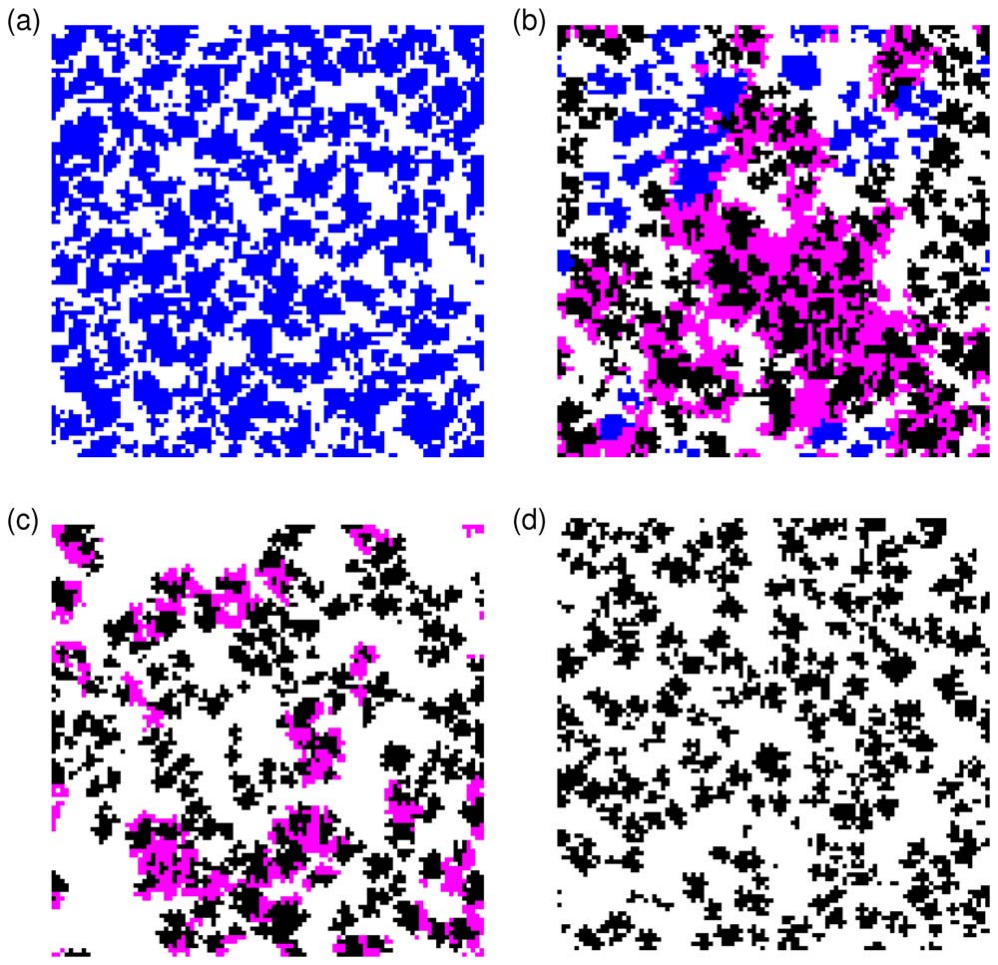
Example 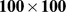 lattice configurations for 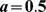 and 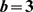 (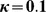). (Non-advertising) defectors (D) are white, advertising defectors (DA) magenta, non-advertising cooperators (C) blue and advertising cooperators black (CA).(a) 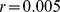 coexistence of C and D. (b) 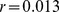 Coexistence of all four types. (c) 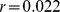 Coexistence of D, DA and CA. The spatial organisation of the clusters makes it quite clear that advertising defectors invade clusters of advertising cooperators, but are beaten by non-advertising defectors. Advertising cooperators can invade non-advertising defectors, resulting in a cyclical game. (d) 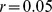 Coexistence of D and CA.

In the following, I verify and extend the above arguments by numerical simulation experiments. The panels in Fig. 1 show data for the dependence of the concentrations of the four strategies in the population on the dilemma strength 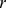 for 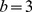 and 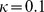. For better visualization, the panels of Fig. 2 illustrate some typical snapshots of arrangements of cooperators and defectors for interesting parameter regions and give support for the above arguments about the cyclical dominance of advertising and non-advertising defectors and advertising cooperators. Fig. 1 explores various regimes of advertising cost parameters 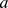. Panel (a) characterizes the regime 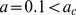 in which advertising is viable for both cooperators and defectors. As a result, only advertising cooperators and defectors survive and the diagram reproduces the known phase diagram with a very low extinction threshold for cooperation at 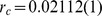 [39]. Similarly, panel (d) gives data for 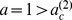, a scenario in which advertising is not viable for both strategies and again the known phase diagram is reproduced.

As argued above, the intermediate interval 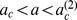 is of more interest, cf. panels (b) and (c). Three observations stand out: i) coexistence of all four strategies appears only possible in a small interval of dilemma strengths below the known extinction threshold 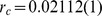. In particular for larger advertising cost, advertsing cooperators only become viable once the dilemma strength exceeds some threshold. Likewise, in the absence of advertising cooperators for low 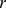, advertising defectors cannot exist and only become viable at the same threshold dilemma strength at which advertising cooperators appear.

**Figure 3:**
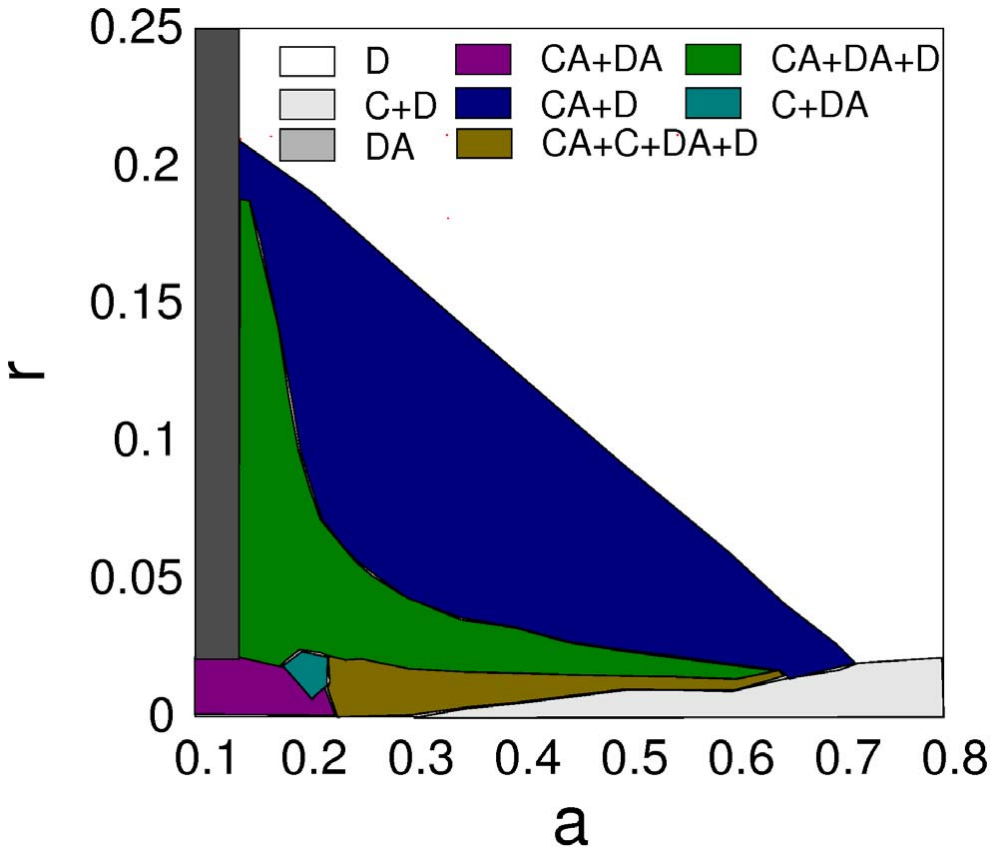
Phase diagram for the entire space of dilemma strengths and advertising costs for 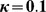 and 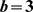.

- Increasing the dilemma strength benefits advertising defectors, provided non-advertising cooperators are still in the population. Once these have died out (for very low dilemma strength), increasing 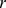 further increases the rates at which advertising defectors invade advertising cooperators and slows down the rate at which advertising cooperators invade non-advertising defectors. The result is a slow decrease in the numbers of advertising cooperators and a strong increase in concentrations of non-advertising defectors which also effect a strong decrease in the numbers of advertising defectors. Hence, further increases in 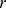 first drive advertising defectors into extinction, resulting in a co-existence regime of advertising cooperators and non-advertising defectors, cf. the phase diagram in the 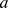-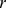 plane in Fig. 3. The extinction of advertising defectors may allow for a recovery in the population of cooperators, but further increases of 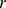 gradually reduce survival chances for advertising cooperators.

**Figure 4:**
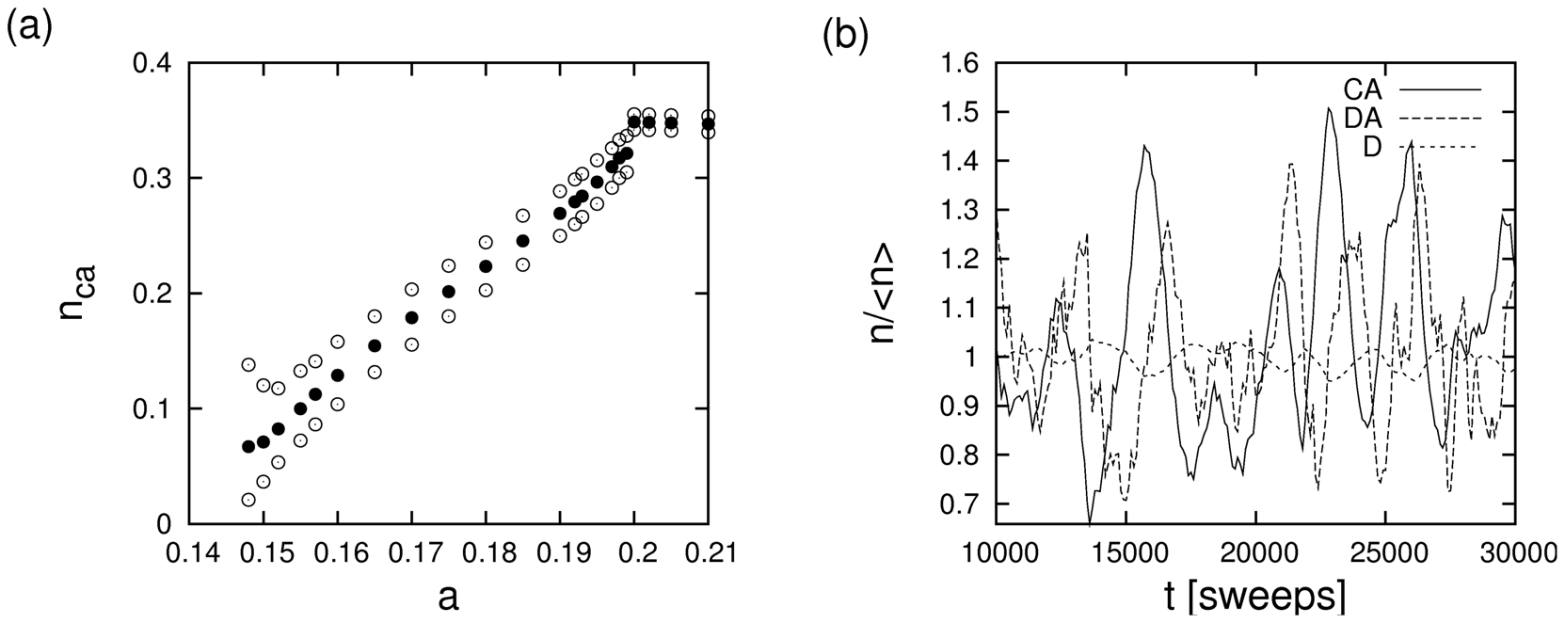
Oscillations in the dynamics of advertising cooperators, advertising defectors and non-advertising defectors in simulations on a 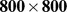 torus for 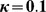 and 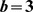. (a) Dependence of the average (solid dots) and maximum and minimum values of the CA population on the cost of advertising. For low cost, amplitudes of the oscillations become very large such that species can go extinct. At 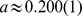 advertising defectors become extinct. This threshold also demarcates the regime of oscillatory dynamics. (b) Timeseries of the evolution of the normalized concentrations of the three species for 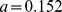. Initial transients have been omitted.

As one would expect from previous work on cyclical dominance between three strategies [38], [39], wave-like patterns and oscillations are found in the regime in which the strategies CA, DA, and D coexist. Some simulation results that demonstrate this are illustrated in Fig. 4. Panel (a) visualizes simulation data for the dependence of the concentration of advertising cooperators and the maxima and minima of oscillations on the cost of advertising. Towards the lower end of cost parameters amplitudes of oscillations are very large. Amplitudes decline when the cost of advertising is increased and advertising defectors decrease in numbers. Eventually, the extinction threshold of advertising defectors at 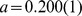 marks the end of the parameter regime in which oscillations can occur. Panel (b) shows some typical timeseries of all three species in the regime that supports oscillations. It should, however, be noted that because invasions at various places will increasingly compensate each other when the system size is increased, the amplitudes of these oscillations is a declining function of system size. This contrasts spatially synchronized patterns that have, e.g., been observed in [40].

- Cyclical dominance and the suppression of advertising defectors at large dilemma strengths replace the competition between cooperators and defectors by a competition between advertising cooperators and non-advertising defectors. The mechanism results in a considerable extension of the range of dilemma strenths for which cooperation can survive. This range is particularly large for small costs of advertising just at the threshold cost given by Eq. (8) and decreases linearly with costs, cf. also the phase diagram for 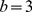 and 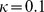 in Fig. 3.

The full phase diagram in the 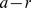 plane given in Fig. 3 shows that regimes exists in which various combinations of the four strategies can co-exist. Several of the transitions between such regimes are discontinuous. One example is the transition 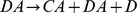 at the sharp cutoff point defined by Eq. (8). Another example is the transition 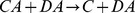 which is the result of an indirect territorial battle of 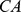 and 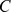, similar to what has been described in the context of adaptive rewarding for public goods games [41], [42]. In the case presented here, the outcome of the competition of 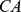 and 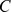 to invade 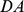 defines the transition point. Similarly, also the transitions 
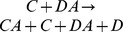
 and 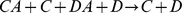 have been found to be discontinuous.

**Figure 5:**
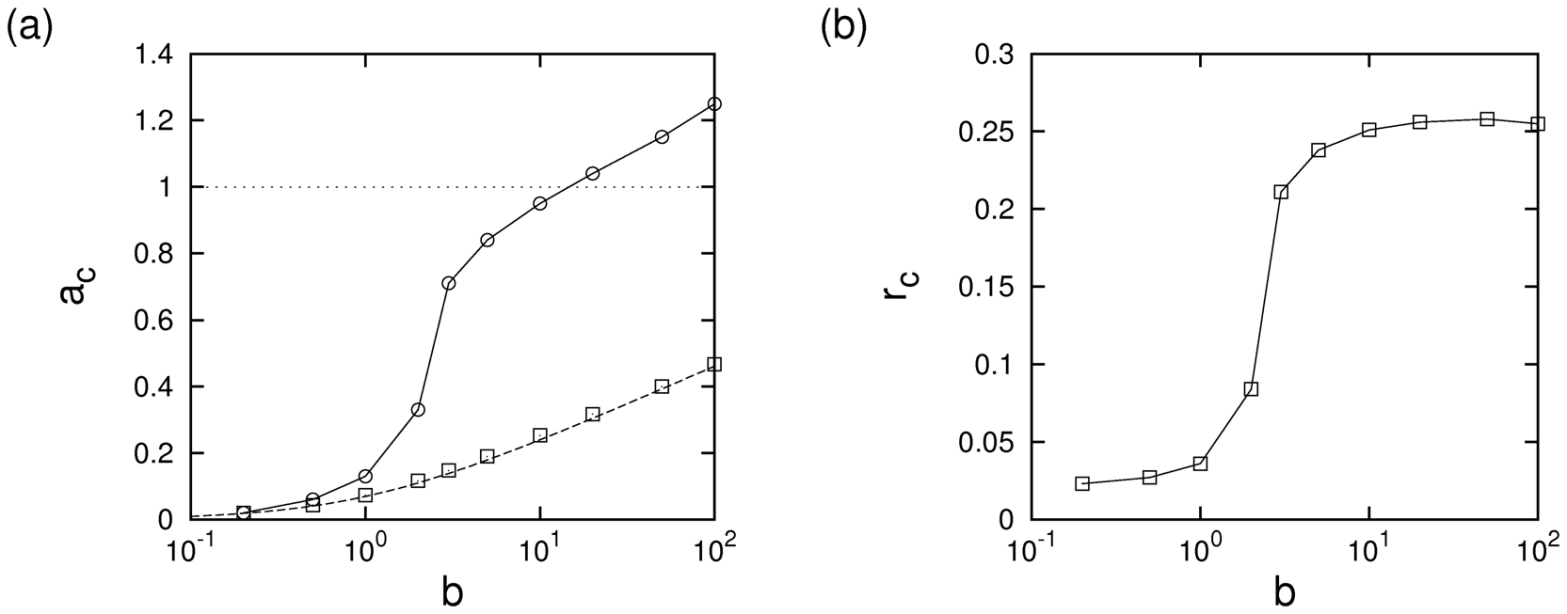
Cost boundaries and maximum support for cooperation. (a) Cost boundaries within which cooperation finds additional support by advertising. Open symbols represent simulation data on a 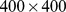 torus using 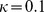. Error bars are about the size of the symbols. The dashed line is plotted using Eq. (8), the solid line is a guide for the eye and the dotted line demarcates the region of advertising costs in which advertising can be socially beneficial (see text). (b) Extinction threshold at which advertising cooperators die out (for the minimum possible cost of advertising).

It is of interest how the size of the cooperation-supporting region depends on the two parameters cost of advertising 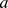 and benefit of advertising 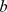 that characterize the coevolutionary advertising game. The panels belonging to Fig. 5 illustrate simulation results addressing this question. In panel (a), maximum and minimum costs required for cooperation to survive are given. One notes, that the lower bound agrees very well with the logarithmic dependence predicted by Eq. (8). In contrast, the upper boundary has a step-like dependence on the benefit of advertising. Whereas a cooperation-supporting range of costs exists for every value of 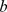, the size of the region increases markedly at 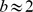. At the same threshold there is also a jump in the range of dilemma strengths at which cooperation can survive, see panel (b). This indicates that cooperation benefits when advertising becomes more effective. However, in finite systems this is not necessarily the case. In particular for large 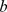 in cost regions in which advertising defectors can survive, amplitudes of the oscillations in cooperator and defector populations can become very large such that advertising cooperators can often go extinct when absorbing boundaries are hit.

### Robustness

How sensitive to changes in model structure is the cooperation-supporting effect of advertising? In this section I will briefly comment on a number of important factors.

First, I note that noise, i.e. an even only small but non-vanishing chance of passing on a strategy that is less successful than the strategy of the focus agent, is essential for the mechanism to operate. In fact, for 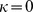 transmission probabilities in Eq. (4) become step-like, strategy propagation becomes deterministic and advertising is never viable. On the other hand, when 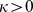 advertising can always support cooperation. Simulations indicate that the support for cooperation is maximized the smaller the amount of noise. In the light of the importance of noise for advertising to be successful, it remains an interesting question whether an equivalent of the underlying mechanism can be found for strategy replication mechanisms other than Fermi-pairwise updating, like e.g. the replicator dynamics.

A second comment is in order about the binary nature of strategies in the present model. One might wonder, if cooperation could be supported if the advertising trait was a continuous variable. Preliminary simulations indicate that this is indeed the case, but the viable amounts of advertising between advertising defectors and cooperators would vary. Detailed results about this model will be reported elsewhere.

Third, outcomes of evolutionary simulations can depend critically on the updating scheme, e.g. whether one chooses synchronous or asynchronous updating [7]. The difference between both schemes tends to be not so important for probabilistic models like the one discussed in this paper. Experiments with a parallel updating scheme show that principal results are robust with regard to the choice of updating scheme. In fact, synchronous updating appears to enhance the regime in which cooperation is supported.

A fourth point worth noting is that the support for cooperation from advertising is not only due to a cyclical dominance mechanism between three strategies. A prototypical phase diagram for not too large benefit of advertising like the one of Fig. 3 will always contain a large region in which the usual competition between cooperators and defectors is replaced by a competition between advertising cooperators and non-advertising defectors. In this regime cooperation can maximally exploit investments into surrounding themselves by cooperators which is a strategy that is not viable for defectors.

One might wonder if the investment into advertising would not obliterate the benefits of cooperation? A quick back of the envelope calculation shows that this is typically not necessarily case. For an estimate of the social benefits of advertising, compare the social payoffs of a pair of advertising cooperators and a pair of non-advertising defectors. In the first case, a group payoff of 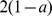 is achieved and in the second case one has a payoff of zero. Hence advertising is overall beneficial if it can lend support to cooperation for 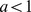. A comparison with Eq. (8) shows that this imposes a limit on 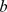 and one obtains the rough estimate of 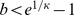 for advertising to be socially beneficial. Fig. 5 illustrates that this estimate is well below the threshold-value of 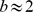 and thus the viable region in 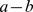 parameter space is rather large and will become the larger the smaller the amount of noise 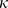 in strategy propagation. In contrast to the above, in the regime 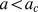 in which advertising is beneficial for both cooperators and defectors advertising is not socially beneficial and may assume the character of an arms race between cooperators and defectors.

A further point worth mentioning is the importance of the mechanism by which advertising and game strategies are inherited. The present model assumes that both strategy components are passed on at the same time and there is no separation of timescales between the spread of the game and the advertising strategies. This is in fact a crucial assumption and some exploratory simulation experiments show that the support for cooperation is reduced markedly if this condition is relaxed.

Last, it is worth remarking that the success of advertising for cooperators is not limited to the two-player version of the prisoner’s dilemma game. Preliminary results show that a very similar mechanism can also operate in the public goods game.

## Discussion

In this paper I have discussed ‘advertising’ as a mechanism by which agents can make a costly investment into faster strategy propagation in evolutionary dilemma games in space. Importantly, both strategy components, advertising and game strategy, are subject to the same evolutionary dynamics and their interplay can co-create dynamic patterns of fast and slow strategy propagators that can sustain cooperation far beyond the regime in which cooperation is supported by network reciprocity in the standard spatial evolutionary game. An important requirement for this extension of the support for cooperation is an appropriate choice of advertising costs. Since cooperators gain support by surrounding themselves with like strategies, advertising can benefit cooperators more than defectors. Hence advertising needs to be so costly that it becomes unviable for defectors, but should be below another threshold that demarcates the maximum benefit for cooperators.

A careful analysis of the costs and benefits of advertising reveals that such a regime always exists, provided that strategy propagation is subject to noise which occasionally allows inferior strategies to be copied. I have discussed that support for cooperation by advertising is robust and advertising can be socially beneficial for the group, provided that the benefits of advertising are not too large.

As a more general aside, advertising essentially introduces a second game on top of the original dilemma situation. The essence of this second game is competition for an accelerated rate of strategy propagation. Considering this game as standalone, advertising could be interpreted as a defect strategy, because it leads to inferior group payoffs compared to non-advertising. However, in the combined game in structured populations a linkage disequilibrium develops. Cooperation in the prisoner’s dilemma game associates with the defect strategy in the advertising game, whereas defect in the prisoner’s dilemma associates with a cooperate strategy in the advertising game. Interestingly, payoffs achieved in the combined game can be larger than in the standalone prisoner’s dilemma game.

The present model serves two purposes. First, by demonstrating that even if subject to the same evolutionary dynamics a trait that marks heterogeneity in strategy spread (i.e. the advertising strategy) and cooperation can co-evolve it addresses a gap in the current literature. Second, by pointing out that costly replication can support cooperation, it may point to a more general mechanism that might lead to some interesting directions for future work.
